# Reports by caregivers of behavioral and psychological symptoms of dementia

**DOI:** 10.1590/S1980-57642008DN10100015

**Published:** 2007

**Authors:** Francisco de Assis Carvalho do Vale, Ricardo Guarnieri, Marcos Liboni, Ari Pedro Balieiro Jr., José Humberto Silva-Filho, Stênio José Correia de Miranda

**Affiliations:** 1 Neurologist, Behavioral Neurology Group, Clinics Hospital of the Ribeirão Preto Faculty of Medicine.; 2 Psychiatrist, Behavioral Neurology Group, Clinics Hospital of the Ribeirão Preto Faculty of Medicine.; 3 Psychologist, Behavioral Neurology Group, Clinics Hospital of the Ribeirão Preto Faculty of Medicine.

**Keywords:** BPSD, behavioral symptoms, psychotic disorders, mood disorders, personality disorders, dementia, caregiver, sintomas comportamentais, transtornos psicóticos, transtornos do humor, transtornos da personalidade, demência, cuidador

## Abstract

Behavioral and Psychological Symptoms of Dementia (BPSD) are relevant since they are frequent and cause distress to caregivers. However, they may not be reported by physicians due to the priority usually attributed to cognitive symptoms.

**Objectives:**

To verify whether BPSD is being systematically investigated by physicians even in specialized settings and whether their records on medical files are accurate.

**Methods:**

Assessment of records on medical files of BPSD reported by caregivers to 182 patients (57.1% men, mean age 67.6±13.5 years) assisted in a tertiary-care behavioral neurology outpatient clinic (BNOC) who also had appointments in other clinics of the same hospital. Alzheimer’s disease (37.9%) and vascular disease (19.2%) were the most frequent causes of dementia.

**Results:**

Report/appointment ratios were 0.58 in BNOC, 0.43 in other neurological, 0.93 in psychiatric and 0.20 in non-neurological, non-psychiatric clinics. BPSD most frequently recorded in BNOC were insomnia, aggressiveness, agitation/hyperactivity, visual hallucinations, apathy, inadequate behavior and ease of crying. Sorted by psychiatrists, categories associated to more BPSD were affect/mood, thought and personality/behavior. affect/mood and sensoperception symptoms were the most frequently reported. Sorted according to Neuropsychiatric Inventory (NPI), categories associated to more BPSD were depression/dysphoria, delusion and apathy/indifference. depression/dysphoria and agitation/ aggression symptoms were the most frequently reported.

**Conclusions:**

BPSD reported by caregivers were very diverse and were not systematically investigated by physicians. Notes in medical files often contained non-technical terms.

## Abstract (pt)

Sintomas Comportamentais e Psicológicos de Demência (SCPD) são relevantes, pois são freqüentes e causam estresse aos cuidadores. Contudo, podem não ser relatados pelos médicos devido à prioridade usualmente atribuída aos sintomas cognitivos.

**Objetivos:**

Verificar que SCPD podem não ser sistematicamente investigados pelos médicos mesmo em ambientes especializados e que seus registros nos prontuários podem ser imprecisos.

**Métodos:**

Avaliação dos registros nos prontuários médicos dos SCPD de relatos de cuidadores de 182 pacientes (57,1% homens, idade média 67,6±13,5 anos) assistidos em um ambulatório de neurologia comportamental (ANCP), que também tiveram consultas em outras clínicas neurológicas, psiquiátricas, não-neurológicas e não-psiquiátricas do mesmo hospital. Doença de Alzheimer (37,9%) e doença vascular (19,2%) foram as causas mais freqüentes de demência.

**Resultados:**

As razões relato/consulta foram 0,58 no ANCP, 0,43 em outros ambulatórios neurológicos, 0,93 em ambulatórios psiquiátricos e 0,20 em outros ambulatórios não-neurológicos e não-psiquiátricos. SCPD mais freqüentemente anotados no ANCP foram insônia, agressividade, agitação/ hiperatividade, alucinações visuais, apatia, comportamento inadequado e choro fácil. Classificados por psiquiatras, as categorias reunindo mais SCPD foram afeto/humor, pensamento e personalidade/comportamento. sintomas de afeto/humor e sensopercepção foram os mais freqüentemente relatados. Classificados de acordo com o Inventário Neuropsiquiátrico (INP), as categorias reunindo mais SCPD foram depressão/disforia, delírio e apatia/indiferença. sintomas de depressão/disforia e agitação/agressão foram os mais freqüentemente relatados.

**Conclusões:**

SCPD relatados pelos cuidadores eram muito diversos e não eram sistematicamente investigados pelos médicos. Anotações nos prontuários eram freqüentemente feitas com termos não técnicos.

Non-cognitive symptoms occurring in dementia patients constitute a major problem for family members and caregivers. As symptoms are frequent and diverse, the term behavioral and psychological symptoms in dementia (BPSD) was proposed by the International Psychogeriatric Association^1^ It serves to designate a variety of symptoms which includes agitation, aggressiveness, apathy, delusions, hallucinations, depression among many others. This heterogeneity reflects different pathophysiologic states of cerebral regions and different underlying psychopathological mechanisms^2^. Despite their interrelationship, behavioral and cognitive symptoms are different and independent to some extent^3,4^.

BPSD are very frequent in dementia patients in both developed and developing countries^5-7^. Although behavioral and psychological symptoms are ubiquitous in all dementias, their frequency and distribution may vary according to type^8^ severity of dementia^9-11^ and ethnic group^12^.

These symptoms have crucial relevance since they are the most important cause of distress to caregivers and family members, usually leading to institutionalization of patients. The magnitude of burden caused to caregivers and its consequent distress depends on symptom severity, type and also ethnicity^9,13-17^. The occurrence of BPSD considerably increases the economic and social costs of dementia management^7,16,18^.

Despite their high occurrence and importance, behavioral and psychological symptoms may not be reported by physicians due to the priority usually attributed to the investigation of the cognitive symptoms in dementia. In this study, we reviewed the reports of BPSD by caregivers in tertiary care outpatient clinics of a teaching hospital according to the physicians' annotations.

The objective of this study was to verify whether BPSD is being systematically investigated by physicians even in specialized settings and check whether their records on medical files are accurate and adequate.

## Methods

We reviewed the medical files of all dementia patients assisted in the Behavioral Neurology Outpatient Clinic (BNOC) during a 3-year period of follow up (1997-1999). BNOC, which has been described elsewhere, is a tertiary care facility of a teaching hospital, the Clinics Hospital of the Ribeirão Preto Faculty of Medicine (CHFMRP)^19^. In the BNOC, diagnoses of Alzheimer’s disease, vascular dementias, Lewy body dementia and frontotemporal dementias are made in accordance with internationally accepted criteria^20-23^. In this outpatient clinic, dementia severity is rated using the Clinical Dementia Rating (CDR)^24-27^. The study was approved by CHFMRP Ethics Committee through its branch Section of Medical Files. Due to the nature of the study, it was not necessary seek Informed Consent.

We actively looked up physicians’ annotations in reports on BPSD by their informant caregivers at appointments made in the BNOC. All appointment records of each patient were reviewed. By informant caregivers we assumed those who were present in the appointment and routinely involved with patient care, comprising mostly family members.

Additionally, we sought physicians’ annotations on reports of BPSD by those BNOC patients' informant caregivers during appointments made in other neurological outpatient clinics, psychiatric outpatient clinics and other non-neurological, non-psychiatric outpatient clinics (e.g., general clinic, cardiology, pneumology) of the same hospital. As all patients were BNOC patients, we performed searches in other clinic appointments in the same way they were done in BNOC appointment annotations.

Initially, we took the literal annotation by the physicians in the reports on behavioral and psychological disorders given by the caregivers. Subsequently, these literal terms were transposed by three of the authors (FACV, RG, ML) to a more technical, closer to the psychopathological terminology. In this manner, some 60 reported symptoms were listed. Next, they asked 22 psychiatrists of CHFMRP to sort those symptoms into five categories of disturbances: affect and mood, thought, sensoperception, personality and behavior and other behaviors that did not fit under in any of these categories. Finally, three of the authors (FACV, APBJ, JHSF) managed to sort those symptoms according to the categories of the Neuropsychiatric Inventory (NPI), although this instrument had not been applied to these patients^28,29^.

We present a descriptive analysis of the reports on BPSD by informant caregivers which were annotated by the physicians during the appointments in the BNOC and in other outpatient clinics of a tertiary care, teaching hospital.

## Results

We studied 182 patients (57.1% of male gender), age range 29-93 years (mean age 67.6±13.5 years). The age of onset of dementia symptoms ranged from 26 to 91 years (mean age of 64.7±14.0 years). Alzheimer's disease (AD) was the most frequent cause, accounting for 37.9% of cases (6.0% of those constituting AD associated with vascular dementia). Vascular dementia (VaD) accounted for 19.2% of cases, other non degenerative dementias for 19.1% (6.0% of those were dementia associated with alcoholism), other degenerative dementia for 9.2% (3.3% were dementia with Lewy bodies and 1.6% were frontotemporal dementia), mixed dementias except AD associated with VaD represented 5.8%. The etiology was not clear in 8.8% of cases. Dementia severity was staged as mild in 23.1% of cases, moderate in 34.1% and severe in 42.8%.

Table 1 shows the numbers of appointment records reviewed, the numbers of appointments with reports of BPSD and report/appointment ratios. The frequency of reports on BPSD by caregivers in the appointments, as taken from the annotations by the physicians, varied among the outpatient clinics.

**Table 1:** Records of appointments reviewed in the search for BPSD* reported by caregivers.

	Number of patients	Total of	Appointments	Report/
	attended in OC	appointments	with reports	appointment
Outpatient clinic (OC)	N (%)	in OC	of BPSD	ratio
BNOC†	182 (100.0)	803	469	0.58
Other neurological	92 (50.5)	257	111	0.43
Psychiatric OC	36 (19.8)	248	231	0.93
Other non-neurological, non-psychiatric OC	90 (49.4)	487	96	0.20

* BPSD, behavioral and psychological symptoms of dementia;

† BNOC, behavioral neurology outpatient clinic.

Table 2 lists all sixty BPSD reported by the informant caregivers, sorted into categories according to the psychiatrists and according to NPI.

**Table 2:** BPSD* reported by caregivers sorted into categories according to psychiatrists and to NPI†.

BPSD	Categories by psychiatrists	NPI
Adynamia	Affect and mood disturbances	Apathy/indifference
Agitation, hyperactivity, restlessness	Personality and behavior disturbances	Agitation/aggression
Alcohol abuse	Personality and behavior disturbances	N/A
Anhedonia	Affect and mood disturbances	Depression/dysphoria
Anxiety	Affect and mood disturbances	Anxiety
Apathy/lack of initiative	Affect and mood disturbances	Apathy/indifference
Arrogance	Personality and behavior disturbances	Irritability/lability
Auditory hallucination	Sensoperception disturbances	Hallucination
Avolition	Affect and mood disturbances	Apathy/indifference
Childish behavior	Personality and behavior disturbances	Disinhibition
Confabulation	Thought disturbances	Delusion
Decreased appetite	Affect and mood disturbances	Appetite/eating change
Decreased libido	Affect and mood disturbances	Depression/dysphoria
Delusion of guilt	Thought disturbances	Delusion
Delusion of jealousy	Thought disturbances	Delusion
Delusion of ruin	Thought disturbances	Delusion
Delusion of theft	Thought disturbances	Delusion
Depression	Affect and mood disturbances	Depression/dysphoria
Ease of crying	Affect and mood disturbances	Depression/dysphoria
Emotional lability	Affect and mood disturbances	Irritability/lability
Euphoria	Affect and mood disturbances	Euphoria/elation
Fear	Affect and mood disturbances	Anxiety
Hipersomnia	Affect and mood disturbances	Night-time behavior
Hopelessness	Affect and mood disturbances	Depression/dysphoria
Idea rumination	Affect and mood disturbances	Depression/dysphoria
Ideas of abandonment	Affect and mood disturbances	Depression/dysphoria
Ideas of death	Affect and mood disturbances	Depression/dysphoria
Ideoaffective dissociation	Affect and mood disturbances	Depression/dysphoria
Illusion	Sensoperception disturbances	Hallucination
Impatience	Affect and mood disturbances	Anxiety
Inadequate behavior	Personality and behavior disturbances	Disinhibition
Increased appetite	Affect and mood disturbances	Appetite/eating change
Increased libido, sexual disinhibition	Affect and mood disturbances	Disinhibition
Indifference	Affect and mood disturbances	Apathy/indifference
Insomnia	Affect and mood disturbances	Night-time behavior
Irritability	Affect and mood disturbances	Irritability/lability
Jocosity (improper laughs and jokes)	Affect and mood disturbances	Disinhibition
Lack of interest (daily activities, hobbies, job, etc)	Affect and mood disturbances	Apathy/indifference
Leave home aimlessly	Other behavior not sorted into previous categories	Aberrant motor behavior
Multiple complaints	Affect and mood disturbances	N/A
Nervousness	Affect and mood disturbances	Irritability/lability
Other (tactile, gustatory, olfactory) hallucinations	Sensoperception disturbances	Hallucination
Other delusional thoughts ("this is not home, I want to go home")	Thought disturbances	Delusion
Persecutory delusion	Thought disturbances	Delusion
Pessimism	Affect and mood disturbances	Depression/dysphoria
Physical aggressiveness	Personality and behavior disturbances	Agitation/aggression
Psychomotor slowness/bradyphrenia	Thought disturbances	Apathy/indifference
Rummage	Other behavior not sorted into previous categories	Aberrant motor behavior
Sadness	Affect and mood disturbances	Depression/dysphoria
Shouting, calling	Other behavior not sorted into previous categories	N/A
Soliloquy	Thought disturbances	Delusion
Solitude	Affect and mood disturbances	Depression/dysphoria
Suicidal attempt	Affect and mood disturbances	Depression/dysphoria
Suicidal ideation	Affect and mood disturbances	Depression/dysphoria
Sundowning	Other behavior not sorted into previous categories	Agitation/aggression
Tachylalia/verbosity	Thought disturbances	Disinhibition
Verbal aggressiveness	Personality and behavior disturbances	Agitation/aggression
Visual hallucination	Sensoperception disturbances	Hallucination
Wandering	Other behavior not sorted into previous categories	Aberrant motor behavior
Withdrawal, isolation, antisocial behavior	Personality and behavior disturbances	Depression/dysphoria

* BPSD, behavioral and psychological symptoms of dementia;

† NPI, neuropsychiatric inventory; N/A, not applicable.

Table 3 shows the number of symptoms sorted into each of five categories by the psychiatrists, the overall number of reports of symptoms in each category and the number of patients with reports of symptoms in each category.

**Table 3:** BPSD* reported by caregivers, sorted by psychiatrists into categories

	Types of BPSD	Reports of BPSD	Patients presenting BPSD
Categories	N (%)	N (%)	N (%^a^)
Affect/mood disturbances	33 (55.0)	1,434 (50.0)	150 (82.4)
Thought disturbances	10 (16.7)	271 (9.5)	80 (44.0)
Personality/behavior disturbances	8 (13.3)	294 (10.3)	67 (36.8)
Sensoperception disturbances	4 (6.7)	796 (27.8)	134 (73.6)
Other behavioral disturbances not sorted	5 (8.3)	72 (2.5)	29 (15.9)
into previous categories			
Total	60 (100.0)	2,867 (100.0)	--

* BPSD, behavioral and psychological symptoms of dementia;

a Percentages in relation to the total of patients studied (182).

Table 4 shows the number of symptoms sorted into each of twelve categories of NPI, the overall number of reports of symptoms in each category and the number of patients with reports of symptoms in each category.

**Table 4:** BPSD reported by caregivers, sorted by the authors into categories of the neuropsychiatric inventory.

Categories	Types of BPSD	Reports of BPSD	Patients presenting BPSD
	N (%)	N (%)	N (%a)
Depression/dysphoria	15 (25.0)	467 (26.3)	80 (44.0)
Delusions	8 (13.3)	241 (8.4)	62 (34.1)
Apathy/indifference	6 (10.0)	258 (9.0)	79 (43.4)
Disinhibition	5 (8.3)	149 (5.2)	59 (32.4)
Agitation/aggression	4 (6.7)	523 (18.2)	111 (61.0)
Hallucination	4 (6.7)	294 (10.3)	67 (36.8)
Irritability/lability	4 (6.7)	199 (6.9)	58 (31.9)
Aberrant motor behavior	3 (5.0)	12 (0.4)	8 (4.4)
Anxiety	3 (5.0)	132 (4.6)	33 (18.1)
Appetite/eating change	2 (3.3)	126 (4.4)	43 (23.6)
Night-time behavior	2 (3.3)	307 (10.7)	80 (44.0)
Euphoria/elation	1 (1.7)	9 (0.3)	4 (2.2)
N/A (not applicable)	3 (5.0)	150 (5.2)	34 (18.7)
Total	60 (100.0)	2,867 (100.0)	-

BPSD, Behavioral and Psychological Symptoms of Dementia; aPercentages in relation to the total of patients studied (182).

On the whole, we observed that these symptoms were not systematically investigated by the physicians in the course of various appointments. Also, annotations were often inaccurate and frequently written in non-technical, lay terms.

BPSD reported by the informant caregivers most frequently annotated by physicians in BNOC appointments were insomnia (8.38%), physical aggressiveness (8.30%), agitation/hyperactivity (7.71%), visual hallucinations (6.69%), apathy (6.35%), inadequate behavior (5.42%) and ease of crying (4.83%). These percentage figures represent report frequencies of each symptom in relation to the overall number of reports. BPSD least annotated by physicians were: ideoaffective dissociation, increased libido, multiple complaints, illusion, arrogance, leave home aimlessly, rummaging and sundowning (0.08% each); hopelessness, ideas of abandonment, pessimism, rumination of ideas, other (tactile, gustatory, olfactory) hallucinations and childish behavior (0.17% each); anhedonia, lack of interest (daily activities, hobbies, job, etc.), solitude, delusion of jealousy and delusion of theft (0.25% each); euphoria and tachylalia/verbosity (0.34% each); indifference, jocosity (improper laughs and jokes) and suicidal attempt (0.42% each).

## Discussion

The percentage distribution of the etiology does not reflect that of populational studies, since this is a casuistry taken from tertiary care outpatient clinics of a teaching hospital. In this setting, the frequency of cases are mostly made up of referrals by primary and secondary care physicians and by physicians from other specialty outpatient clinics in the hospital, as was described elsewhere for the BNOC^19^. A possible bias in the discussion of our data is that it lacks the level of education of the informant caregivers, and education may be a factor influencing the perception and report of behavioral and psychological symptoms. In a previous paper, mean schooling of the BNOC dementia patients was 2.96±3.17 years (19) and hence it might also be inferred that the level of education of caregivers was also low.

Concerning the frequency of reports of BPSD by caregivers in the appointments, as taken from the physicians annotations in the medical files, the report/appointment ratios varied among different clinics. The highest report/ appointment ratio (0.93) occurred in psychiatric outpatient clinics probably due to the nature of symptoms. However, even in this context, 7.0% of appointments lacked annotation of BPSD, possibly because they went uninvestigated by the physicians. By taking 0.93 as a “gold standard” in this casuistry, the report/appointment ratio in BNOC (0.58) might be considered low since it is a specialized neurological clinic attending dementia patients. The ratio was even lower in other neurological clinics (0.43) but that could be accounted for the occurrence of other relevant neurological symptoms to be reported in their appointments. The lowest ratio occurred in other non-neurological, non-psychiatric outpatient clinics, as one might expect. All these outpatient clinics are practices with medical residences, and trainee physicians may not be aware of the importance of investigating BPSD even in neurological settings.

The most generally reported BPSD by informant caregivers in the BNOC annotated by the physicians were insomnia, physical aggressiveness and agitation/hyperactivity. Rates of BPSD vary according to setting and ascertainment^6^, studies highlighting different symptoms as being the most frequently reported, namely depression^5,11^, aberrant motor behavior^13^ and apathy^4,14^.

The least reported symptoms, all with less than 0.50% of occurrence each, were ideoaffective dissociation, increased libido, multiple complaints, illusion, arrogance, leave home aimlessly, rummage, sundowning, hopelessness, ideas of abandonment, pessimism, rumination of ideas, other (tactile, gustatory, olfactory) hallucinations, childish behavior, anhedonia, lack of interest (daily activities, hobbies, job, etc.), solitude, delusion of jealousy, delusion of theft, euphoria, tachylalia/verbosity, indifference, jocosity (improper laughs and jokes) and suicidal attempt. Rates of the least reported behavioral and psychological symptoms vary due to the same reasons as for the most reported ones, studies have indicated euphoria^10,13,14^ hallucinations and disinhibition^14^ as being amongst the least reported.

According to the sorting of BPSD by psychiatrists, the category under which most symptoms were assigned was affect/mood disturbances (55.0 of symptoms), followed by thought disturbances (16.7%) and personality/behavior disturbances (13.3%). Affect/mood disturbance symptoms were also the most frequently reported (50.0% of the overall number of reports) but here the secondplaced category is sensoperception disturbances (27.8%).

In reference to the sorting of BPSD reported by informant caregivers according to NPI categories, one must stress that it was merely an attempt to add information and enrich the discussion because this instrument was not applied to the patients of this casuistry. Presently, the BNOC dementia patients have been assessed using the NPI, to be reported in a coming paper. Several BPSD reported could be sorted into more than one category; however the authors chose the most suitable categories by taking into consideration the structure and the set of questions of the NPI. Also, some BPSD reported did not fit under any NPI category (alcohol abuse, multiple complaints, shouting/calling). The categories assigned most symptoms were depression/dysphoria (25.0% BPSD reported), delusions (13.3%) and apathy/indifference (10.0%). with regard to the frequency of reports of symptoms, the most important categories were depression/ dysphoria (26.3% of the overall number of reports) and agitation/aggression (18.2%).

In this casuistry, BPSD reported by informant caregivers were more diverse. They were not systematically investigated by the physicians. Despite being an important cause of distress for family members and caregivers, such symptoms were not always properly described in the medical files whereas the annotations were also inaccurate and written with the use of non-technical terms. On the other hand, this highlights the need for systematically looking out for behavioral and psychological symptoms when examining patients with cognitive disorders and dementias, perhaps possible with the aid of appropriate questionnaires and inventories^4,5,16^.
